# Full Characterisation of Heroin Samples Using Infrared Spectroscopy and Multivariate Calibration

**DOI:** 10.3390/molecules29051116

**Published:** 2024-03-01

**Authors:** Eric Deconinck, Sybrien Lievens, Michael Canfyn, Peter Van Campenhout, Loic Debehault, Lies Gremaux, Margot Balcaen

**Affiliations:** 1 Sciensano, Scientific Direction Chemical and Physical Health Risks, Service of Medicines and Health Products, J. Wytsmanstraat 14, B-1050 Brussels, Belgium; sybrien.lievens@vub.be (S.L.); michael.canfyn@sciensano.be (M.C.); peter.vancampenhout@sciensano.be (P.V.C.); loic.debehault@sciensano.be (L.D.); 2 VUB, Faculty of Sciences and Bio-Engineering, Department Chemistry, Analytical, Environmental and Geo-Chemistry, Pleinlaan 2, B-1050 Brussels, Belgium; 3 Sciensano, Scientific Direction Epidemiology and Public Health, Service Lifestyle and Chronic Diseases, J. Wytsmanstraat 14, B-1050 Brussels, Belgium; lies.gremaux@sciensano.be (L.G.); margot.balcaen@sciensano.be (M.B.)

**Keywords:** adulterants, ATR-IR, chemometrics, diacetylmorphine, diluents, mobile detection approaches

## Abstract

The analysis of heroin samples, before use in the protected environment of user centra, could be a supplementary service in the context of harm reduction. Infrared spectroscopy hyphenated with multivariate calibration could be a valuable asset in this context, and therefore 125 heroin samples were collected directly from users and analysed with classical chromatographic techniques. Further, Mid-Infrared spectra were collected for all samples, to be used in Partial Least Squares (PLS) modelling, in order to obtain qualitative and quantitative models based on real live samples. The approach showed that it was possible to identify and quantify heroin in the samples based on the collected spectral data and PLS modelling. These models were able to identify heroin correctly for 96% of the samples of the external test set with precision, specificity and sensitivity values of 100.0, 75.0 and 95.5%, respectively. For regression, a root mean squared error of prediction (RMSEP) of 0.04 was obtained, pointing at good predictive properties. Furthermore, during mass spectrometric screening, 10 different adulterants and impurities were encountered. Using the spectral data to model the presence of each of these resulted in performant models for seven of them. All models showed promising correct-classification rates (between 92 and 96%) and good values for sensitivity, specificity and precision. For codeine and morphine, the models were not satisfactory, probably due to the low concentration of these impurities as a consequence of acetylation. For methacetin, the approach failed.

## 1. Introduction

The principles of harm-reduction initiatives are deeply embedded in the EU Drugs Strategy and Action Plan (2021–2025) [1]. These initiatives aim at reducing the health-related risks linked to the use of illicit drugs by applying a more pragmatic approach. One of these initiatives is drug checking, by which users of psychoactive substances can submit their samples for chemical analysis and receive timely feedback about composition and possible risks related to the samples submitted [2,3,4,5,6]. Furthermore, these services might also provide consumption rooms, where people can self-administer drugs in a protected and hygienic environment, overseen by qualified staff [7]. This is especially valuable for the use of hard drugs or injected products such as heroin. In the latter case, the combination of a chemical analysis of the product and use in the supervised environment of the user centrum can significantly reduce the risk of incidents or even lethal consequences. 

However, the effective testing of illicit drug samples in the context of drug checking relies on the possibility of low-cost and fast analytical approaches. The latter results in the fact that drug checking services often rely on colour tests and spectroscopic approaches such as ultraviolet–visible, Fourier-transform infrared and Raman spectroscopy [2,8,9,10]. The colour test kits are often used as a first step in the analysis, and even if they are purely presumptive in nature, they can be considered as fairly accurate in identifying compounds or mixtures of compounds, especially when a battery of tests is used [8]. Although these techniques have many advantages, they are all limited when it comes to the identification of multiple drugs in one sample, purity determination or the detection of unknown molecules. Although some spectroscopic instruments come with software allowing deconvolution, and thus the detection or identification of several compounds in a mixture, this remains limited to the main compounds, while impurities or low-dosed adulterants will be missed [9,11]. This is of particular interest for heroin, which is also called 3,6-diacetyl morphine and represents about 8% of the world’s drug seizures. In its pure form, it is a white powder with a bitter taste. However, heroin is rarely encountered as such and often contains many impurities and adulterants, resulting in colours ranging from white to dark brown. The wide variety in purity poses the greatest risk when using heroin, due to the difficulty in dosing and thus the risk of (lethal) overdoses [12,13]. For illustration, a Serbian study showed a variety in purity in their sample set from 1.7 to 58.8% *w*/*w* [13]. Diluents that are often encountered in these samples are inert powders like chalk, flour, talcum powder and glucose, but also pharmacological active compounds like acetaminophen and caffeine [12,13]. A whole range of impurities might be present, originating from the production process. Two special cases in the context of adulteration have become important in the last decade. The first is the well documented fentanyl crisis, wherein heroin (and also cocaine) is adulterated with fentanyl [14,15]. This is done by the producers and without the knowledge of the consumers. The second is the trend in which substances are intentionally mixed with heroin. An example is a product sold under the name “Speedball”, which consists of cocaine mixed with heroin [16]. Fentanyl and its analogues caused the fentanyl crisis, mainly in North America, where they were intentionally added to heroin and cocaine samples [14,15]. These fentanyls are considered new psychotropic substances and are a major concern due to their prevalence, diversity and potency [14,15]. It is especially their potency that is responsible for the numerous deaths caused by this crisis [14,15]. They are present in the samples as low-dosed adulterants, and therefore can be missed during analysis with the techniques available from drug checking services [14,15]. Speedball, which consists of the simultaneous injection of cocaine and heroin, recently showed an increased popularity. The mixture, causing the feeling of a high to be more intense and to last longer, elevates the health risks for the user, since heroin acts as a depressant, while cocaine is a stimulant, causing opposite side effects [16,17,18]. 

As illustrated before, heroin is a complex mixture of active and inert compounds and represents a challenge for analysis with the techniques often available at consumption rooms or drug checking services. In general, heroin samples are analyzed using gas or liquid chromatography paired with mass spectrometry (GC/LC–MS), possibly followed by other techniques for purity determination or quantification of certain adulterants [8,9]. From the techniques available at the majority of the drug checking services, infrared spectroscopy (IR) shows the highest potential, especially when it is used together with multivariate calibration techniques [12,13,19,20,21,22,23]. The combination of infrared spectroscopy, both mid- and near IR, and multivariate calibration was already applied in a plethora of fields, ranging from illegal medicines [24] and cosmetics [25], through e-liquids [26,27] and food analysis [28,29,30] to the analysis of illicit drug samples. In the context of the latter, it was also applied for the analysis of heroin. The applications using a combination of IR and multivariate calibration for the analysis of illicit drug samples were recently reviewed [19]. Unfortunately most of these applications focus on the identification and quantification of heroin [12,20,21], while the applications taking into account some diluents and adulterants only did so for standard mixtures or self-prepared samples [13,22,23]. 

Therefore, this paper explores the possibilities of mid-IR spectroscopy coupled with multivariate calibration for the identification and quantification of heroin, as well as the detection of 10 adulterants and impurities in real samples previously analyzed with standard laboratory techniques, namely GC–MS and LC–diode array detection (LC–DAD) analysis. In total, 125 samples were collected and analysed using GC–MS and LC–DAD. Next, the mid-IR spectra were collected and the data were analysed. As a first step, data exploration was performed using principal component analysis (PCA) followed by qualitative and quantitative modeling by partial least squares (PLS). Both PCA and PLS are often used in combination with spectroscopic data and were already applied in the analysis of illicit drugs and more specifically for heroin samples [19].

## 2. Results

### 2.1. Characterisation of the Samples

All collected samples were screened for the presence of illicit drugs, adulterants and impurities using the previously mentioned GC–MS method (Section 3.3.2). For the samples positive for heroin, the dosage/purity was consequently determined using the UHPLC method described in Section 3.3.3. Table 1 gives an overview of the results obtained for the full characterisation of the 125 samples.

Overall, it was observed that only seven samples could not be identified as containing heroin, from which two were positive for acetaminophen and caffeine. The most encountered adulterants or impurities in the heroin samples were caffeine, acetylcodeine, diacetamate, acetaminophen, papaverine, morphine and noscapine. Morphine, codeine, noscapine and papaverine are common alkaloids present in the samples, since these phenanthrenes (morphine and codeine) and benzylisoquinolines (noscapine and papaverine) are components of opium and frequently remain as impurities after extraction [31,32]. It has to be noted that only 23% of the heroin samples in our sample set contained codeine. This is probably due to the fact that the majority of the present codeine was acetylated during the extraction process used during heroin production. Next to these expected impurities and adulterants, three samples were found to be adulterated with cocaine, one with bromazepam, a benzodiazepine, and one with ketamine. The purity of the 118 samples identified as heroin varied between 0.21% m/m and 56.53% m/m, with a median purity of 11.01% m/m and a mean purity of 13.01% m/m. 

In our sample set, no fentanyl or fentanyl analogues could be detected. 

**Table 1:** Overview of the results obtained by the GC–MS and UHPLC analysis of the 125 samples in the sample set. Presence (+) and absence (−) of contaminants and adulterants and results of the assay of 3,6-Diacetyl Morphine.

	Purity Heroin (%)	3,6-Diacetyl Morphine	Aceta- Minophen	Diacetamate	Caffeine	Codeine	Morphine	Acetyl- Codeine	6-Monoacetyl Morphine	Papaverine	Noscapine	Methacetin
S1	0.00%	−	−	−	−	−	−	−	−	−	−	−
S2	1.01%	+	+	+	+	+	+	+	+	−	+	−
S3	13.90%	+	+	+	+	+	+	+	+	+	+	+
S4	17.44%	+	+	+	+	+	+	+	+	−	+	+
S5	14.19%	+	+	+	+	+	+	+	+	−	+	−
S6	12.78%	+	+	+	+	+	+	+	+	+	+	+
S7	10.80%	+	+	+	+	+	+	+	+	+	+	+
S8	19.32%	+	+	+	+	+	+	+	+	+	+	+
S9	9.56%	+	+	+	+	+	+	+	+	+	+	+
S10	0.00%	−	−	−	−	−	−	−	−	−	−	−
S11	0.00%	−	−	−	−	−	−	−	−	−	−	−
S12	12.09%	+	+	+	+	+	+	+	+	+	+	−
S13	11.90%	+	+	+	+	+	+	+	+	+	+	−
S14	9.92%	+	+	+	+	+	+	+	+	+	+	+
S15	10.08%	+	+	+	+	+	+	+	+	+	+	−
S16	10.48%	+	+	+	+	+	+	+	+	+	+	−
S17	10.28%	+	+	+	+	+	+	+	+	+	+	−
S18	9.63%	+	+	+	+	−	+	+	+	+	+	−
S19	11.86%	+	+	+	+	+	+	+	+	+	+	−
S20	12.40%	+	+	+	+	−	+	+	+	+	+	−
S21	22.72%	+	+	+	+	−	+	+	+	+	+	−
S22	12.02%	+	+	+	+	+	+	+	+	+	+	−
S23	11.04%	+	+	+	+	+	+	+	+	+	+	+
S24	8.98%	+	+	+	+	−	+	+	+	+	+	−
S25	10.22%	+	+	+	+	−	+	+	+	+	+	−
S26	9.98%	+	+	+	+	+	+	+	+	+	+	−
S27	10.04%	+	+	+	+	+	+	+	+	+	+	−
S28	15.05%	+	+	+	+	−	+	+	+	+	+	−
S29	16.74%	+	+	+	+	+	+	+	+	+	+	−
S30	11.84%	+	+	+	+	+	+	+	+	+	+	−
S31	9.99%	+	+	+	+	−	+	+	+	+	+	−
S32	12.29%	+	+	+	+	−	+	+	+	+	+	−
S33	56.53%	+	−	+	+	−	−	+	+	+	+	−
S34	9.60%	+	+	+	+	−	+	+	+	+	+	−
S35	10.43%	+	+	+	+	−	+	+	+	+	+	−
S36	17.55%	+	+	+	+	+	+	+	+	+	+	−
S37	17.22%	+	+	+	+	+	+	+	+	+	+	−
S38	9.51%	+	+	+	+	+	+	+	+	+	+	−
S39	14.44%	+	+	+	+	−	+	+	+	+	+	−
S40	14.32%	+	+	+	+	+	+	+	+	+	+	−
S41	17.17%	+	+	+	+	−	+	+	+	+	+	−
S42	13.76%	+	+	+	+	+	+	+	+	+	+	−
S43	10.01%	+	+	+	+	−	+	+	+	+	+	−
S44	12.00%	+	+	+	+	−	+	+	+	+	+	−
S45	13.76%	+	+	+	+	−	+	+	+	+	+	−
S46	9.76%	+	+	+	+	−	+	+	+	+	+	−
S47	9.89%	+	+	+	+	−	+	+	+	+	+	−
S48	0.21%	+	+	−	+	−	−	−	+	−	−	−
S49	36.61%	+	−	−	−	−	−	+	+	+	+	−
S50	17.37%	+	+	+	+	+	+	+	+	+	+	−
S51	17.77%	+	+	+	+	+	+	+	+	+	+	−
S52	13.19%	+	+	+	+	−	+	+	+	+	+	−
S53	9.17%	+	+	+	+	−	+	+	+	+	+	−
S54	6.16%	+	+	+	+	−	+	+	+	+	+	−
S55	9.29%	+	+	+	+	−	+	+	+	+	+	−
S56	10.41%	+	+	+	+	−	+	+	+	+	+	−
S57	20.23%	+	+	+	+	−	+	+	+	+	+	−
S58	35.69%	+	+	+	+	−	+	+	+	+	+	−
S59	14.95%	+	+	+	+	−	+	+	+	+	+	−
S60	10.06%	+	+	+	+	−	+	+	+	+	+	−
S61	18.76%	+	+	+	+	−	+	+	+	+	+	−
S62	10.49%	+	+	+	+	−	+	+	+	+	+	−
S63	10.71%	+	+	+	+	−	+	+	+	+	+	−
S64	13.72%	+	+	+	+	−	+	+	+	+	+	−
S65	10.08%	+	+	+	+	−	+	+	+	+	+	−
S66	10.16%	+	+	+	+	−	+	+	+	+	+	−
S67	10.16%	+	+	+	+	−	+	+	+	+	+	−
S68	10.20%	+	+	+	+	−	+	+	+	+	+	−
S69	0.42%	+	+	+	+	−	−	+	+	−	+	−
S70	9.96%	+	+	+	+	−	+	+	+	+	+	−
S71	5.31%	+	+	+	+	−	+	+	+	+	+	−
S72	10.97%	+	+	+	+	−	+	+	+	+	+	−
S73	7.61%	+	+	+	+	−	+	+	+	+	+	−
S74	9.50%	+	+	+	+	−	+	+	+	+	+	−
S75	18.35%	+	+	+	+	−	+	+	+	+	+	−
S76	8.18%	+	+	+	+	+	+	+	+	+	+	−
S77	10.97%	+	+	+	+	−	+	+	+	+	+	−
S78	10.82%	+	+	+	+	−	+	+	+	+	+	−
S79	10.91%	+	+	+	+	−	+	+	+	+	+	−
S80	10.71%	+	+	+	+	−	+	+	+	+	+	−
S81	17.74%	+	+	+	+	−	+	+	+	+	+	−
S82	17.61%	+	+	+	+	−	+	+	+	+	+	−
S83	0.00%	−	−	−	−	−	−	−	−	−	−	−
S84	12.01%	+	+	+	+	−	+	+	+	+	+	−
S85	12.16%	+	+	+	+	−	+	+	+	+	+	−
S86	9.85%	+	+	+	+	−	+	+	+	+	+	−
S87	9.03%	+	+	+	+	−	+	+	+	+	+	−
S88	0.00%	−	−	−	−	−	−	−	−	−	−	−
S89	12.33%	+	+	+	+	−	+	+	+	+	+	−
S90	7.13%	+	+	+	+	−	+	+	+	+	+	−
S91	17.55%	+	+	+	+	−	+	+	+	+	+	−
S92	1.82%	+	+	+	+	−	−	+	+	+	+	−
S93	4.93%	+	+	+	+	−	+	+	+	+	+	−
S94	11.84%	+	+	+	+	−	+	+	+	+	+	−
S95	11.62%	+	+	+	+	−	+	+	+	+	+	−
S96	11.43%	+	+	+	+	−	+	+	+	+	+	−
S97	6.66%	+	+	+	+	−	+	+	+	+	+	−
S98	10.16%	+	+	+	+	−	+	+	+	+	+	−
S99	14.31%	+	+	+	+	−	+	+	+	+	+	−
S100	22.35%	+	+	+	+	−	+	+	+	+	+	−
S101	11.31%	+	+	+	+	−	+	+	+	+	+	−
S102	4.92%	+	+	+	+	−	+	+	+	+	+	+
S103	4.30%	+	+	+	+	−	+	+	+	+	+	−
S104	12.45%	+	+	+	+	−	+	+	+	+	+	−
S105	11.34%	+	+	+	+	−	+	+	+	+	+	−
S106	47.78%	+	+	+	+	−	−	+	+	+	+	−
S107	10.16%	+	+	+	+	−	+	+	+	+	+	−
S108	10.02%	+	+	+	+	−	+	+	+	+	+	−
S109	14.41%	+	+	+	+	−	+	+	+	+	+	−
S110	14.20%	+	+	+	+	−	+	+	+	+	+	−
S111	13.01%	+	+	+	+	−	+	+	+	+	+	+
S112	20.96%	+	+	+	+	−	+	+	+	+	+	−
S113	44.67%	+	+	+	+	−	+	+	+	+	+	−
S114	0.00%	−	+	−	+	−	−	−	−	−	−	−
S115	0.00%	−	+	−	+	−	−	−	−	−	−	−
S116	19.68%	+	+	+	+	−	+	+	+	+	+	−
S117	1.97%	+	+	+	+	−	+	+	+	+	+	−
S118	5.74%	+	+	+	+	−	+	+	+	+	+	−
S119	9.60%	+	+	+	+	−	+	+	+	+	+	−
S120	8.81%	+	+	+	+	−	+	+	+	+	+	−
S121	23.67%	+	+	+	+	−	+	+	+	+	+	−
S122	10.52%	+	+	+	+	−	+	+	+	+	+	−
S123	14.21%	+	+	+	+	−	+	+	+	+	+	−
S124	14.22%	+	+	+	+	−	+	+	+	+	+	−
number positive samples		117	117	115	118	29	111	116	117	112	116	10

### 2.2. Unsupervised Analysis

**Figure 1:**
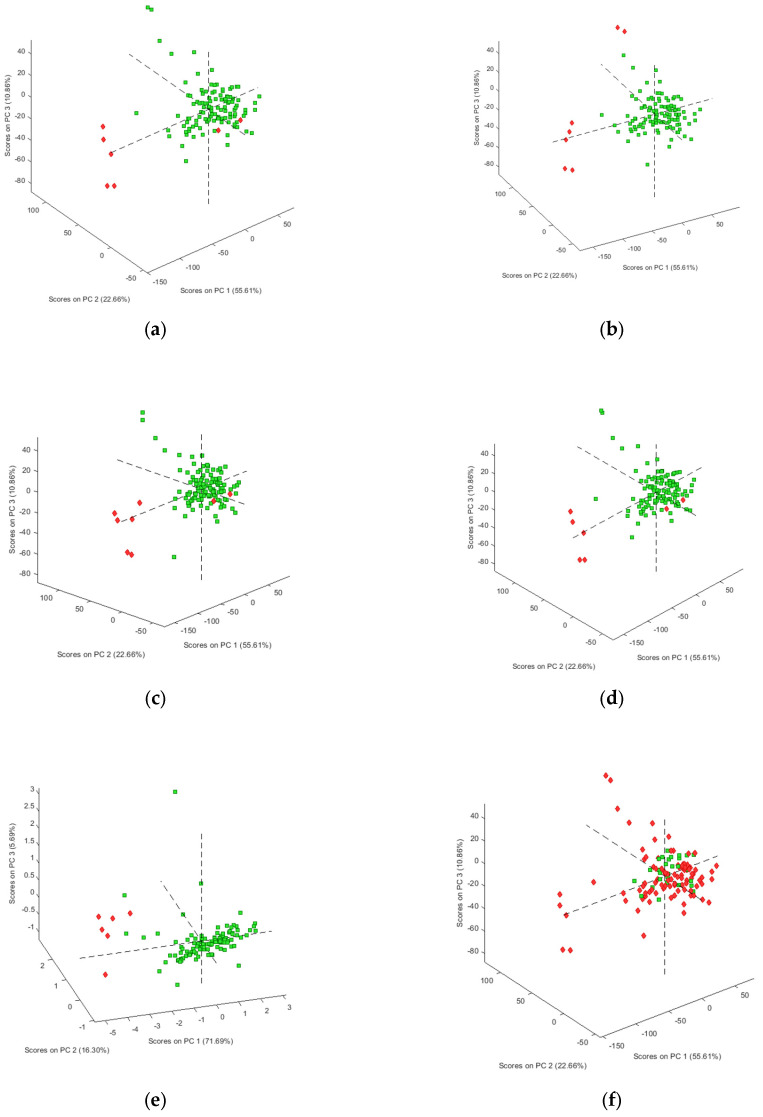

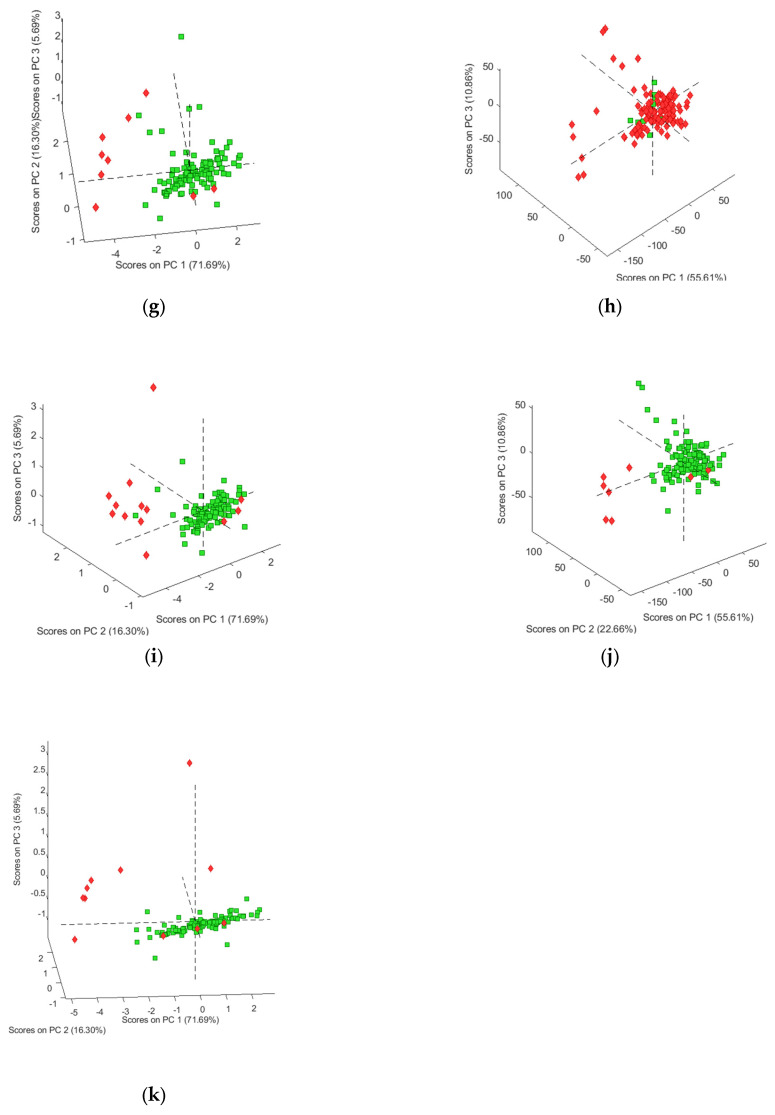
Three-dimensional PCA score plots (negative samples red, positive samples green) for (**a**) heroin (autoscaling); (**b**) acetaminophen (autoscaling); (**c**) acetylcodeine; (**d**) acetylmorphine (autoscaling); (**e**) caffeine (mean centering); (**f**) codeine (autoscaling); (**g**) diacetamate (mean centering); (**h**) methacetin (autoscaling); (**i**) morphine (autoscaling); (**j**) noscapine (autoscaling); (**k**) papaverine (mean centering).

**Figure 2:**
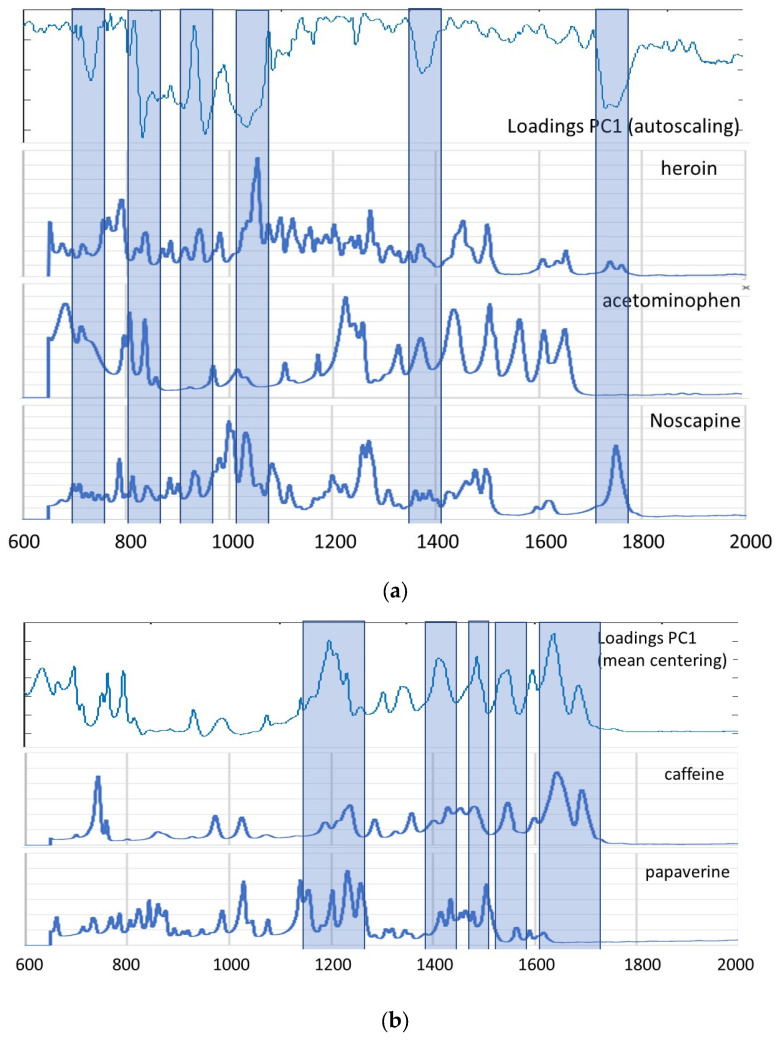
(**a**) Loading plot obtained after autoscaling with the MIR reference spectra for heroin, acetaminophen and noscapine and the indication of the correspondences; (**b**) loading plot obtained after mean centering with the MIR reference spectra for caffeine and papaverine and the indication of the correspondences.

As a first step, the spectral data for the 125 samples in our sample set were explored using the unsupervised data analysis technique PCA, though the figures were interpreted for each compound separately, reflecting the clustering of negative and positive samples for heroin and each of the found adulterants and impurities. The analysis was performed four times using autoscaling, SNV, mean centering and the second derivative as data pre-treatment, respectively. Figure 1 shows the best PCA score plots obtained for each of the targeted molecules. Figure 1a clearly shows a clustering of the negative samples for heroin, except for two samples. This is probably due to the fact that these samples contain caffeine and acetaminophen, which makes them more closely related to the positive samples. This hypothesis is supported by the fact that these samples were also clustered when the presence of acetaminophen and caffeine was used for interpretation of the PCA results (Figure 1b,e). Results similar to those for heroin were obtained for acetylcodeine, mono-acetylmorphine, diacetamate and noscapine (Figure 1c,d,g,j), where each time the two heroin-negative samples containing acetaminophen and caffeine were not clustered with the other negative samples. On the other hand, for the presence of codeine (Figure 1f) and methacetin (Figure 1h), no clustering could be observed separating the negative and positive samples. For morphine (Figure 1i) and papaverine (Figure 1k), clustering could be observed in, respectively, 9 of the 13 and 8 of the 12 negative samples, and this in the plane defined by PC1 and PC2. No clear explanation could be given for the negative samples clustered with the positive samples. In order to check that the observed clusterings are indeed related to the presence of the different targeted components, the loadings were investigated for each of the PCA analyses performed. Figure 2 shows the loading plots for the first PC, obtained after autoscaling (Figure 2a) (best clustering for heroin, acetaminophen, acetylcodeine, mono-acetylmorphine, morphine and noscapine) and mean centering (Figure 2a) (best clustering for caffeine, diacetamate and papaverine). The loadings reflect the importance of the spectral regions for the definition of the latent variable, and so also for the observed clustering. The importance can be either negative or positive. If highly positive or negative loadings can be related to specific signals of the IR spectrum of the targeted molecule, this indicates that the distinction between positive and negative samples is indeed due to the presence of this compound. For some of the molecules, the loadings could be related to specific areas in the IR spectrum. Figure 2a,b therefore also shows the reference spectra for the components where there was a clear correspondence between the loadings and the absorbances. These correspondences prove that the clustering is clearly related to the differences in infrared spectra between the samples and thus to the composition, which is in fact a mixture of different compounds. The loadings on the higher PCs display less correspondence, which is to be expected. 

Overall, it could be concluded that the recorded spectral data relate quite well to the presence of heroin and the different encountered adulterants and impurities, justifying the application of supervised PLS analysis to create classification and regression models. 

### 2.3. Supervised Analysis

A duplex algorithm was applied to select an external test set containing 25 of the 125 samples. Since it was decided in this research to use only real-life samples, the data set is not equilibrated between negative and positive samples. Therefore, after each selection of a test set, it was verified whether the ratio between negative and positive samples in the test set reflected the ratio of the whole sample set. The ratios of positive to negative samples for each of the targeted compounds can be found in Table 2. For all compounds, qualitative models were constructed using PLS–Discriminant Analysis (DA), and an overview of the modelling and validation parameters can be found in Table 2. 

Since only 3,6-diacetylmorphine or heroin was quantified, only for this compound was PLS applied to become a regression model for the purity of the heroin samples. 

#### 2.3.1. Heroin

**Figure 3:**
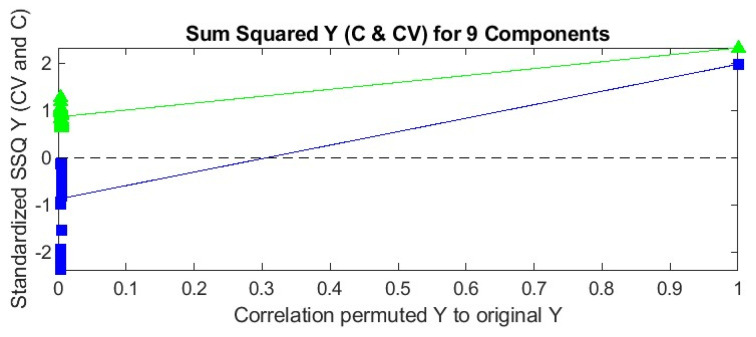
SSQY versus Y-block correlation plot for the PLS–DA model obtained for heroin (blue: results for cross validation; green: results for self-prediction (calibration)).

For heroin, the best qualitative model was obtained using nine PLS factors and the second derivative as data pre-treatment method. This model showed a correct classification rate (ccr) for cross validation of 98.99% and for the test set of 96.00%, both corresponding to one sample that was incorrectly classified as a positive sample. Both misclassifications correspond to the samples negative for heroin but positive for acetaminophen and caffeine. It seems that these samples are too similar to the positive heroin samples to be distinguished based on their mid-IR spectrum, as was also the case during unsupervised analysis. Good values for sensitivity and precision are obtained for this model. The lower values for specificity are only due to the very low number of negative samples in the sample set, but as said before, the model only showed one false-positive sample both in the training and test set. Although the good predictive results for the external test set indicate a lack of overfitting of the model, a permutation test was performed for the selected PLS–DA model. The test was performed both on the self-prediction residuals and on the cross-validated residuals [33]. The pairwise Wilcoxon signed-rank test, the pairwise sign test and the randomization *t*-test were used to prove that the obtained model is not significantly different from one created by randomly shuffling the response variables (classes), and this at the 5% probability level. All statistics showed values between 0.000 and 0.012, so less than 0.05, indicating no significant difference and thus a low risk of overfitting [33]. A visual interpretation of the permutation test is possible based on the plot of sum squared Y (SSQY) versus Y-block correlation (Figure 3). The plot shows the SSQY values for both self-prediction (green) and cross-validation (blue) as a function of the Y-block correlation. The model is unlikely to overfit, since the cross-validated and self-predicted values are relatively close to each other, but significantly different from the results for the non-permuted Y-block (on the far right side of the plot) [33]. 

**Figure 4:**
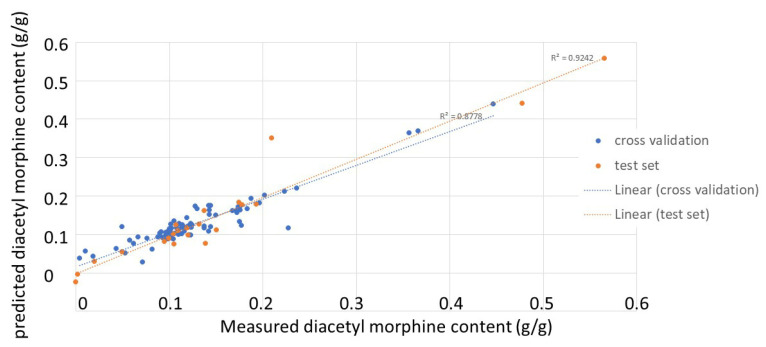
Correlation plots between real and predicted purity values for the heroin-positive samples (blue: results for cross validation; orange: results for the external test set).

After the removal of the negative samples, a PLS model was built using the purities determined by UHPLC as response variables. Similarly, the different pre-treatment methods were explored, and the best model was obtained using SNV and seven PLS factors. This model showed a root mean squared error of calibration (RMSEC) of 0.02 and a determination coefficient for calibration of 0.91. Figure 4 shows the correlations between the real and predicted values for the samples, both for cross-validation (internal validation) and for the test set (external validation). The determination coefficients were 0.88 and 0.92, respectively. With root mean squared error of cross-validation (RMSECV) and root mean squared error of prediction (RMSEP) values of 0.03 and 0.04, respectively, one can say that this PLS model was able to closely relate the spectral data to the purity values of the heroin samples and was able to provide good predictions for new samples, both in cross-validation and with an external test set. 

#### 2.3.2. Acetaminophen

Similar results were obtained for the classification of the different samples according to the presence of acetaminophen. The best performing model consisted of two PLS factors using SNV as data pre-treatment. The model showed ccr values of 98.99% and 96.00% for cross-validation and the test set, respectively, which correspond to one sample classified as a false positive in cross-validation and one sample considered a false negative in the test set. Similar to the heroin analysis, very promising results were obtained for sensitivity, precision and specificity, taking into account the limited number of true negative samples in the sample set. No clear explanation could be found for both misclassifications. Possibly, the false-negative sample could contain a low amount of acetaminophen, but this should be confirmed by a quantitative analysis, which was not performed due to the limited amount of sample available. The permutation tests showed a maximum value of 0.038 for the randomization *t*-test, pointing to a low risk of overfitting (at the 95% level), as all values were lower than 0.05.

#### 2.3.3. Acetylcodeine

The best model obtained for the presence of acetylcodeine in the samples is the PLS–DA model using five PLS factors and SNV as data pre-treatment. The model showed a ccr for cross validation of 98.99% and one for the test set of 96.00%. Both in the training and in the test set, one sample was classified as a false positive. The two samples misclassified here were the same as the ones misclassified by the heroin model, namely the one positive for acetaminophen and caffeine but negative for heroin. This seems logical, as basically the same spectral data are used for all compounds, and the presence of acetylcodeine as an impurity is highly related to the presence of heroin. Once more, very good results were obtained for specificity, sensitivity and precision, taking into account the low number of negative samples in the sample set. The permutation tests showed a maximum value of 0.007 for the randomization *t*-test, pointing to a low risk of overfitting (at the 95% level), as all values were lower than 0.05. 

#### 2.3.4. Mono-Acetyl Morphine

Highly similar results were obtained with the optimal model for mono-acetyl morphine using the second derivative and nine PLS factors. Here also, ccr values of 98.99% and 96.00%, respectively, were obtained for cross-validation and the test set, corresponding to the two samples negative for heroin but positive for acetaminophen and caffeine, which were also considered here to be false positives. The same trend was also seen in the values for sensitivity, specificity and precision. The permutation tests showed a maximum value of 0.019 for the randomization *t*-test, pointing to a low risk of overfitting (at the 95% level), as all values were lower than 0.05.

#### 2.3.5. Caffeine

For caffeine, the optimal model was obtained with eight PLS factors and the second derivative and showed cross-validation and test set ccr values of 98.99% and 96.00%, respectively. In cross-validation, there was one false-positive sample, while in the test set one false-negative sample could be observed. No clear explanation for these misclassified samples could be found. It could be a low dosage in the case of the false negative, or could just be due to random modelling errors. Sensitivity, specificity and precision were also acceptable for this model. The permutation tests showed a maximum value of 0.015 for the randomization *t*-test, pointing to a low risk of overfitting (at the 95% level), as all values were lower than 0.05. 

#### 2.3.6. Codeine

Codeine proved to be quite difficult to model, probably due to the low number of positive samples in the sample set and the low concentrations. In all the analysed samples, codeine is present as a low-level impurity, since normally the majority of the codeine present is acetylated and thus present under the form of acetylcodeine. This was also shown by the relative intensities between the different signals in the GC–MS chromatogram. 

The best-performing PLS model was the one using autoscaling as data pre-treatment method and 15 PLS factors. Ccr values of 88.82% for cross-validation and of 80.00% for the external test set were obtained. Taking a closer look at the predictions shows that in cross-validation, seven samples were considered false positives, while nine samples were false negatives. Similarly, for the test set, three false positives and two false negatives could be observed. It could be observed that all misclassified samples had a quite low purity for heroin, but a real explanation could not be found. We have to consider these as random modelling errors, caused by the low concentration of this impurity in the heroin samples, which can be considered as complex mixtures. 

The inadequate results obtained for codeine is also reflected in the values for sensitivity, specificity and precision. 

#### 2.3.7. Diacetamate

The classification of the samples according to the presence of diacetamate was found best with a PLS model constructed with 14 PLS factors and the second derivative of the mid-IR spectra. Ccr values of 97.98% and 96.00% were obtained for cross-validation and the test set, representing two false-positive samples during cross-validation and one for the test set. The false positive for the test set and one of the misclassified samples in cross-validation correspond to the two samples negative for heroin and positive for caffeine and acetaminophen, which were also badly clustered during the exploratory PCA analysis. Sensitivity, specificity and precision values showed promising values, considering the low number of negative samples. The permutation tests showed a maximum value of 0.005 for the randomization *t*-test, pointing to a low risk of overfitting (at the 95% level), as all values were lower than 0.05. 

#### 2.3.8. Methacetin

For methacetin, no significant model could be found. All models classified all samples as negative, both in cross-validation and in the test set. Here, it could only be concluded that methacetin cannot be detected using an approach combining mid-IR spectroscopy and PLS–DA. 

#### 2.3.9. Morphine

As for codeine, the modelling of the presence of morphine was more challenging, probably due to the same issue that morphine is present as a low-dosed impurity, since the majority of the present morphine was acetylated during the production/extraction process. 

The best model for morphine was obtained using SNV and three PLS factors. Ccr values of 96.97% and 88.00% were obtained, respectively, for cross-validation and the test set, representing three false-positive samples for each. No sound explication for the misclassified samples could be found. The challenge to modelling is also reflected by the low values for specificity of the model, meaning that at least 50% of the negative samples is predicted as a false positive. During the interpretation of the values, the very low number of morphine negative samples should be kept in mind. 

#### 2.3.10. Noscapine

A PLS model using SNV and five PLS factors was found to be the most suitable for the classification of the samples according to the presence of noscapine. The model has a ccr for cross-validation of 98.98% and one of 96% for the external test set, representing a false-positive sample in both. These two misclassified samples corresponded again to the two samples negative for heroin but positive for acetaminophen and caffeine. Considering the low number of noscapine-negative samples, satisfying values were obtained for sensitivity, specificity and precision. The permutation tests showed a maximum value of 0.012 for the randomization *t*-test, pointing to a low risk of overfitting (at the 95% level), as all values were lower than 0.05. 

#### 2.3.11. Papaverine

Autoscaling and five PLS factors resulted in the best-performing model for papaverine, another alkaloid impurity, originating from opium. Validation of the model resulted in a ccr of 97.98% for cross-validation and 92.00% for the external test set. These values correspond to two false-positive samples in, respectively, cross-validation and external validation. Two of the false-positive samples were the ones found negative for heroin but positive for acetaminophen and caffeine, and the two others were heroin samples with purities of 14 and 17%, which were positive for the other alkaloid impurities originating from opium, but which seemed to have amounts of papaverine lower than the detection limits of the GC–MS screening method. Nevertheless, the model showed good results for sensitivity, specificity and precision. 

The permutation tests showed a maximum value of 0.007 for the randomization *t*-test, pointing to a low risk of overfitting (at the 95% level), as all values were lower than 0.05. 

## 3. Methods and Materials

### 3.1. Standards and Samples

The heroin reference standard was purchased from Lipomed AG (Arlesheim, Switzerland). 

In total, 125 heroin samples were collected in the period between February and May 2023. The samples were collected by three different harm-reduction services, also called grassroot organisations. These organisations are in close contact with the drug users and build strong and confident relationships with them. First, the organisation informed the users through face-to-face contact about the different goals of collecting the samples, and once they were willing to participate, a consent form was signed and a questionnaire was filled in about the context of epidemiologic studies, which is outside of the scope of this paper. A heroin sample of 100 mg was collected in an Eppendorf and uniquely labelled. As an incentive, participants were paid to compensate for the sample. 

### 3.2. Sample and Standard Preparation

For the qualitative analysis using GC–MS, the samples were first ground, and then about 1 mg was dissolved in 1.5 mL of methanol absolute HPLC grade (Biosolve B.V.; Valkenswaard, The Netherlands) in a GC vial (Agilent Technologies, Santa Clare, CA, USA). All manipulations of the samples were performed under a laminar flow hood. The vials with the solutions were vortexed for 20 to 30 s. 

For the quantitative analysis using LC–DAD, about 25 mg of the sample was brought into a 5 mL volumetric flask and diluted with a solvent consisting of a 90/10 mixture of a 0.1% formic acid (98–10%, Merck, Darmstadt, Germany) solution in water and acetonitrile HPLC grade (Biosolve B.V., Valkenswaard, The Netherlands). The samples were sonicated for 25 min, after which they were diluted five times and filtered through a 0.22 μm mixed cellulose ester syringe filter (BGB Analytik Benelux, Harderwijk, The Netherlands).

The calibration standards were prepared in a similar way. About 10 mg of the heroin reference standard was brought in a 20 mL volumetric flask and diluted with the same solvent as the samples. After sonication, the stock solution was diluted with a factor 2, 4 and 10 to obtain the calibration standards for the calibration line and check the validity of the measurements. 

### 3.3. Data Acquisition

#### 3.3.1. FT-Mid-IR

A Nicolet iS10 FTIR (ThermoFisher Scientific, Waltham, MA, USA), equipped with a Smart iTR accessory and a deuterated triglycine sulfate (DTGS) detector, was used to collect the mid-IR spectra for the samples. The Smart iTR accessory (attenuated total reflectance accessory) uses a single-bounce diamond crystal and allows us to measure the spectra immediately from the powdered sample, by deposing a small amount of the sample on the crystal, without further sample preparation. The accessory was calibrated once a week using a polystyrene film. 

Infrared spectra were recorded in the wavenumber range from 4000 to 400 cm^−1^, at a spectral resolution of 4 cm^−1^. Each measurement consisted of 32 co-added scans. Spectral data were treated using the OMNIC Software version 8.3 (Thermo Scientific, Madison, WI, USA). Between the different samples, the crystal was cleaned using a soft tissue soaked with methanol, and after drying in ambient air, a blank measurement was performed to check the crystal for contamination and carry-over using the absorbance limits for contamination defined by the European Directorate for the Quality of Medicines and HealthCare (EDQM) [34]. Every hour, a background spectrum against air was measured as well. 

#### 3.3.2. GC–MS

Qualitative GC–MS analysis was performed using an Agilent 7890A GC-system (Agilent Technologies, Santa Clare, CA, USA) equipped with an Agilent 7683B Series injector and paired with an Agilent 5975C Mass Selective Detector (single quadrupole). Hardware control, data acquisition and data handling were done using the Agilent Masshunter and Data analys^®^ software version 10.0. Chromatographic separation was achieved using an Agilent J&W VF-5ms capillary column (40 m × 0.25 mm; 0.25 μm) with a temperature gradient that started at 80 °C, which was held for two minutes, followed by a gradient at a rate of 15 °C per minute until a temperature of 280 °C was reached. This temperature was held for 17 min, resulting in a total runtime of about 32.3 min. The injection volume was set at 1 μL and helium was used as carrier gas at a constant flow rate of 1.5 mL/min. The injector was operated in split mode (ratio 1:10) and the temperatures of the injection port, the ion source, the quadrupole and the interface were set at 250 °C, 230 °C, 150 °C and 280 °C, respectively. Mass data were recorded in full scan mode. 

The mass spectra of the signals of interest were compared to the reference spectra in the eNIST20 Mass Spectral Library. A match factor above 85% was considered as reliable. If lower, the peaks were manually integrated to confirm the result. 

#### 3.3.3. High Pressure Liquid Chromatography–Diode Array Detection (LC–DAD)

The quantitative analysis to determine the purity of the heroin samples was performed on an Acquity UPLC system (Waters, Milford, CO, USA) consisting of a binary pump, column oven, temperature-regulated autosampler and a diode array detector (DAD). The separation of 3,6-diacety morphine was achieved on a Waters Acquity UPLC C18-BEH column (2.1 mm × 100 mm, 1.7 μm) using a 0.1% *v*/*v* formic acid solution in water (A) as aqueous and acetonitrile (B) as organic mobile phase in gradient mode. The gradient started at 95% A, decreasing to 90% A in 10 min, after which 20% A was reached in 5 min and held for 3 min before returning to the initial conditions. The seal wash consisted of 10% methanol in water, the weak wash of a 30% acetonitrile solution in water and the strong wash of an equal mixture of methanol, acetonitrile, isopropanol and water containing 5% formic acid. The flow rate was set at 0.4 mL/min, the injection volume was 10 μL, the column temperature 30 °C, the autosampler temperature 15 °C and the detection wavelength 280 nm. 

### 3.4. Data Pre-Processing

**Figure 5:**
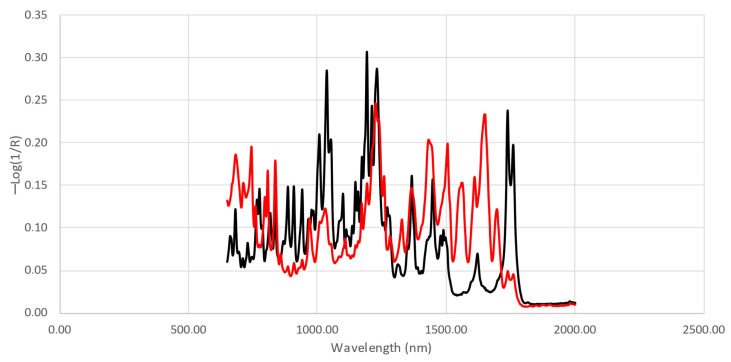
Examples of mid-IR spectra for a low-purity (red) and a high-purity sample (black).

For the chemometric analysis, the so-called fingerprint region, defined by the pseudo-absorbance (−log(1/R)) obtained between 2000 and 650 cm^−1^, was selected. Figure 5 shows an example of a mid-IR spectrum for a sample with low (10%) and high purity (56%). Although clear differences can be observed for two of the extreme samples, differences become smaller with the changes in purity. Small differences due to the presence of impurities and adulterants could not be differentiated visually. Therefore, chemometrics will be necessary to extract the information of interest from the spectral data and to discriminate between the samples based on the different adulterants and impurities and on the purity of 3,6-diacetyl morphine. 

Before the application of chemometric techniques, the pseudo-absorbance spectra were pre-treated to eliminate or reduce variations introduced into the data by external sources other than the sample itself. Several pre-treatment techniques were explored. First, two scaling techniques were applied, namely autoscaling and mean centering. These techniques allow us to correct differences in scale within the data, which could influence the selection of the variables or the importance of some variables during modelling. Further, standard normal variate (SNV) was one of the explored pre-treatment techniques. SNV is a normalisation procedure eliminating the variation in the data due to the measurement itself, e.g., differences in path length, scattering effects, variations in the detector, etc. [35]. Finally, the second derivative was applied. The latter technique removes the background from the spectra and accentuates the spectral framework, and therefore highlights the differences between spectra. The derivative was calculated using the Savitzky–Golay method [36] with a second-order polynomial and a window size of 17. 

All chemometric models need to be validated, and therefore, there is a need for a validation or an external test set consisting of samples/data that were not used for building the models. Hence, an external test set comprising roughly 20% of the complete sample set was selected using the Duplex algorithm [37]. This algorithm is based on a pairwise selection of samples. It starts with the selection of two samples in the data space, with the highest Euclidean distance between them for a first set. The next two samples with the highest Euclidean distance are selected for the second set. This procedure continues by iteratively selecting sample pairs for the first and the second set, until the predefined number of samples is reached in the second set, the test set. The first set and the remaining samples will be used as the training set. Before applying multivariate calibration techniques, it was checked that the ratio of positive/negative samples in the test set was similar to the ratio in the complete sample set (qualitative models). For the quantitative models, it was made sure that the test set covered the whole range of purities in the sample set. 

### 3.5. Principal Component Analysis (PCA)

PCA is a feature reduction technique, allowing the visual representation of high-dimensional data by defining new latent variables, which are defined as linear combinations of the manifest variables. These latent variables are called principal components (PC) and are defined in such a way that they represent the highest variance in the data (PC1) or the highest remaining variation (further PCs). This results in the fact that the different calculated PCs are orthogonal to each other by definition. In PCA, the scores are the projection of the samples on the different PCs and are a measure of the similarity between samples, while the loadings show the respective contribution of the manifest variables to a given PC [35]. 

In this study, PCA is used as an exploratory technique before supervised modelling. 

### 3.6. Partial Least Squares (PLS)

PLS is by the most popular multivariate calibration technique and is in fact a supervised projection technique, similar to PCA. The technique also defines new latent variables, called PLS factors (PLS), as linear combinations of the manifest variables, calculated to represent the highest (PLS1) or remaining (higher PLS factors) co-variance between the data and a response. PLS is applied for regression purposes and used in this study to model the purity of the heroin samples. PLS–DA is an adapted form of PLS allowing classification modelling and thus the use of categorical responses. In this study, PLS–DA is used to create binary classification models based on the recorded IR spectra for the identification of heroin samples and the detection of the 10 different adulterants and impurities encountered during the GC–MS analysis of the samples in our sample set. 

During the calculation, the optimal number of PLS factors to be included in the models was selected based on 10-fold cross-validation. The validation of the different models was based on the root mean squared error of cross-validation (RMSECV, internal validation) for the training set and the root mean squared error of prediction (RMSEP, external validation) for the selected test set. For the regression models (PLS), the coefficients of determination between real and predicted values were calculated as well, for both training and test set. RMSECV and RMSEP were expressed as correct classification rates (ccr) in the case of classification models (PLS–DA), and the sensitivity, specificity and precision were calculated as additional features. 

### 3.7. Software

Data processing and modelling were performed using Matlab version R2020b (The Mathworks, Natick, MA, USA) with the PLS-toolbox v8.9.2 (Eigenvector Research, Inc., Manson, WA, USA). 

## 4. Discussion and Conclusions

In this project, we attempted to characterise “street level” heroin samples based on the presence and the purity level of heroin, as well as the present adulterants and impurities encountered in the sample set, based on mid-IR spectroscopy and PLS modelling, requiring only pulverisation of the samples under investigation. As mentioned previously, infrared spectroscopy and PLS modelling were already applied before in this context for the qualitative and quantitative analysis of 3,6-diacetyl morphine in heroine samples [19]. However, previous studies were limited by a small number of samples, or employed only self-prepared samples and targeted only 3,6-diacetyl morphine [19]. To our knowledge, this study is the first using models based solely on spectra from real-life street samples, including the variety present in a representative set of samples circulating in Belgium, and represents a first step towards a practical approach in the context of onsite testing for harm reduction. It is also the first study in which the combination of spectroscopy and multivariate calibration has been explored for the identification of the encountered adulterants and contaminants. 

It may be shown that the presented approach is able to identify heroin samples and perform a semi-quantitative analysis on 3,6-diacetyl morphine. The models showed a classification error of 4% for identification and a RMSEP for regression of 0.04, with a determination coefficient of 0.92 for the external test set. Based on these two models, harm-reduction services could be able to analyse the heroin samples of people who used drugs without destroying their samples, and thus potentially prevent complications or overdose. One of the requirements to do so is the integration of the model in the vendor software, allowing automatic interpretation without the need of expert knowledge in spectroscopy and chemometrics. 

Next to the analysis of the heroin content, it was also attempted to create models for the adulterants and impurities encountered during the standard analysis of the samples. In total, 10 different adulterants and impurities were modelled and, except for codeine, morphine and methacetin, performant models could be obtained. The inability of the presented approach to model the presence of morphine and codeine is probably due to the fact that these molecules are present in very low amounts, as most of these compounds originating from opium itself are acetylated during the extraction and production process. For methacetin, the approach failed completely. This could also be due to the low concentration of this adulterant in the samples, or simply the fact that the models were unable to extract the information related to this molecule from the spectrum. Heroin samples should be considered as complex mixtures and, therefore, the matrix and the other adulterants and impurities might mask the signal of methacetin in the mid-IR spectrum. In general, it should also be mentioned that our research was limited by the very low amount of negative samples encountered in our sample set. However, the samples collected reflect the real situation, as they all originate from people who use drugs, giving insight into street heroin. A regular update of these models is of prime importance if the approach is to be implemented routinely; this includes gathering new samples in order to update the model for changes in matrices, adulterants, samples and so on. It is plausible that the continuous update of the models will remove the limitations of this paper by including more and more negative samples sold as heroin. 

As NIR spectroscopy might also be available at harm-reduction services, this technique could also be explored in a similar approach, especially for the adulterants that were not able to be modelled based on mid-IR data. A drawback here is the fact that NIR often requires more material and the spectra are less specific. The latter have already been shown to have an influence on the qualitative modelling of, e.g., MDMA [38] and illicit drugs, presented in the form of white powders [39]. The other spectroscopic technique often mentioned for onsite testing of illicit drugs is Raman spectroscopy. However, to our knowledge, no direct Raman spectroscopic approach was described in the literature for the analysis of heroin samples [19]. This is probably due to the complex nature of the heroin matrix and the inconvenience of fluorescence, a phenomenon well known in Raman spectroscopy. Also, these spectra are less specific than the ones of mid-IR, and Raman spectroscopy suffers from low sensitivity. These disadvantages could be partly solved by applying surface-enhanced Raman spectroscopy (SERS), allowing increased sensitivity with several factors, which is already applied for the detection of fentanyl analogues in heroin samples [40,41,42,43] and detection of heroin and its metabolites in human samples [44,45,46]. Although SERS is a promising emerging technique, the more extensive sample preparation and its destructive character make it perhaps less suited for onsite testing in a harm-reduction context, especially since personnel in these centers often do not have an analytical background. 

To conclude, it can be stated that the approach combining mid-IR spectroscopy and PLS modelling is promising for the total characterisation of heroin samples and could be a valuable asset to harm-reduction initiatives. Depending on the samples, the approach could also be broadened to other adulterants, e.g., fentanyl analogues, as was demonstrated by Tobias et al. [47]. The advantage of working with models based on real-life samples is that the real-life variability within the samples is incorporated in the models, allowing more performant and robust models. On the other hand, models can only be built for compounds that occur in the sample set and, thus, compounds that are not part of the approach can be missed. For the latter, it would therefore also be important to always compare the recorded spectrum of the samples to a spectral library with reference spectra containing other compounds of interest, in order to enhance the detection of adulterants not part of the modelling approach. 

## Figures and Tables

**Table 2:** Modelling parameters for qualitative PLS–DA models.

Compound	Nr. Training Samples (Ratio Positive/ Negative)	Nr. Test Set Samples (Ration Positive/ Negative	Data Pre-Treatment	Nr. PLS Factors	Ccr for Calibration (%)	Ccr for Cross Validation (%)	Ccr for Prediction (%)	Sensitivity (%) CV-Test Set	Specificity (%) CV-Test Set	Precision (%) CV-Test Set
Heroin	100 (97/3)	25 (21/4)	2nd derivative	9	98.99	98.99	96.00	100.00–100.00	66.67–75.00	98.98–95.45
Acetaminophen	100 (97/3)	25 (21/4)	SNV	2	100.00	98.99	96.00	100.00–95.45	66.67–100.00	98.98–100.00
Acetyl codeine	100 (97/3)	25 (20/5)	SNV	5	98.99	98.99	96.00	100.00–100.00	66.67–80.00	98.98–95.00
Mono-acetylmorphine	100 (97/3)	25 (21/4)	2nd derivative	9	98.99	98.99	96.00	100.00–100.00	66.67–75.00	98.98–95.23
Caffeine	100 (97/3)	25 (22/3)	2nd derivative	8	100.00	98.99	96.00	100.00–95.45	66.67–100.00	98.98–100.00
Codeine	100 (24/73)	25 (5/20)	Autoscaling	15	92.93	82.83	80.00	62.50–60.00	90.41–85.00	68.18–50.00
Diacetamate	100 (96/4)	25 (20/5)	2nd derivative	14	98.99	97.98	96.00	100.00–100.00	50.00–80.00	97.96–95.23
Methacetine	100 (6/94)	25 (4/21)	-	-	-	-	-	-	-	-
Morphine	100 (94/6)	25 (21/4)	SNV	3	96.97	96.97	88.00	100.00–100.00	50.00–25.00	96.90–87.50
Noscapine	100 (97/3)	25 (20/5)	SNV	5	98.99	98.99	96.00	100.00–100.00	66.67–80.00	98.98–95.23
Papaverine	100 (95/5)	25 (19/6)	Autoscaling	5	97.98	97.98	92.00	100.00–100.00	97.89–89.47	97.94–90.48

## Data Availability

Data are contained within the article.
